# Stress hyperglycemia ratio and long‐term prognosis in patients with acute coronary syndrome: A multicenter, nationwide study

**DOI:** 10.1111/1753-0407.13400

**Published:** 2023-05-03

**Authors:** Guyu Zeng, Ying Song, Zheng Zhang, Jingjing Xu, Zhenyu Liu, Xiaofang Tang, XiaoZeng Wang, Yan Chen, Yongzhen Zhang, Pei Zhu, Xiaogang Guo, Lin Jiang, Zhifang Wang, Ru Liu, Qingsheng Wang, Yi Yao, Yingqing Feng, Yaling Han, Jinqing Yuan

**Affiliations:** ^1^ National Clinical Research Center for Cardiovascular Diseases, State Key Laboratory of Cardiovascular Disease, Fuwai Hospital, National Center for Cardiovascular Diseases Chinese Academy of Medical Sciences and Peking Union Medical College Beijing China; ^2^ Department of Cardiology The First Hospital of Lanzhou University Lanzhou China; ^3^ Department of Cardiology Peking Union Medical College Hospital, Chinese Academy of Medical Sciences and Peking Union Medical College Beijing China; ^4^ Department of Cardiology General Hospital of Northern Theater Command Shenyang China; ^5^ Department of Cardiology Peking University Third Hospital Beijing China; ^6^ Department of Cardiology, The First Affiliated Hospital Zhejiang University School of Medicine Hangzhou China; ^7^ Department of Cardiology Xinxiang Central Hospital Xinxiang People's Republic of China; ^8^ Department of Cardiology The First Hospital of QinHuangDao Qinhuangdao China; ^9^ Department of Cardiology Guangdong Provincial People's Hospital Guangzhou People's Republic of China

**Keywords:** acute coronary syndrome, coronary artery disease, long‐term prognosis, stress hyperglycemia ratio, 急性冠状动脉综合征, 冠状动脉疾病, 远期预后, 应激性高血糖比值

## Abstract

**Background:**

Stress hyperglycemia ratio (SHR), a novel biomarker of stress hyperglycemia, was proved to be a reliable predictor of short‐term adverse outcomes in patients with acute coronary syndromes (ACS). However, its impact on long‐term prognosis remained controversial.

**Methods:**

A total of 7662 patients with ACS from a large nationwide prospective cohort between January 2015 and May 2019 were included. SHR was calculated by the following formula: SHR = admission glucose (mmol/L)/(1.59 × HbA1c [%]−2.59). The primary end point was a major adverse cardiovascular event (MACE) during follow‐up, a composite of all‐cause death, myocardial infarction, and unplanned revascularization. The second end point was the separate components of the primary end points.

**Results:**

During a median follow‐up of 2.1 years, 779 MACE events occurred. After multivariable adjustment, ACS patients with the highest SHR tertile were significantly associated with increased long‐term risks of MACE (hazard ratio [HR] 1.53, 95% confidence interval [CI] 1.24–1.88), all‐cause death (HR 1.80, 95% CI 1.29–2.51) and unplanned revascularization (HR 1.44, 95% CI 1.09–1.91). Although significant associations between the highest SHR tertile and risks of MACE and all‐cause death were assessed in both diabetic and nondiabetic patients, the patterns of risk were different in these two groups.

**Conclusion:**

Elevated SHR was independently associated with a higher risk of long‐term outcomes irrespective of diabetic status, suggesting that SHR was a potential biomarker for risk stratification after ACS.

## Graphical abstract

**Highlights**

- The association between stress hyperglycemia ratio (SHR) and long‐term outcomes was still controversial.
- Based on a large prospective multicenter cohort, we found that high SHR levels were independently associated with increased risk of major adverse cardiovascular events (MACE), all‐cause death, and unplanned revascularization. Associations between high SHR levels and risk of MACE and death remained positive in patients with or without diabetes.
- SHR was a potential biomarker for risk stratification after acute coronary syndromes.

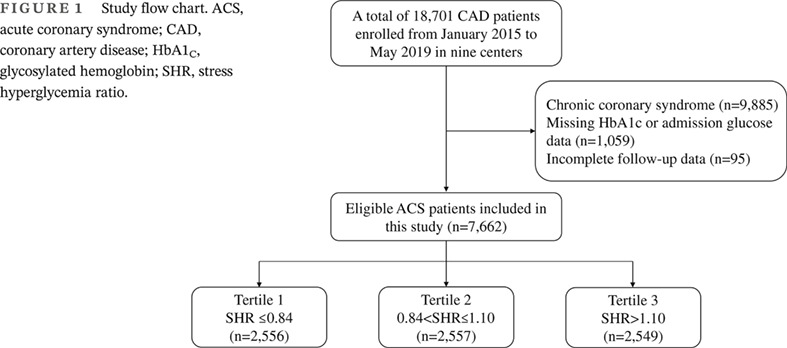

## 摘要

背景:应激性高血糖比率(SHR)是应激性高血糖的一种新型生物标志物, 已被证明是急性冠状动脉综合征(ACS)患者短期不良结果可靠的预测因子。然而, 它对于长期预后的影响仍存在争议。

**方法:**纳入了2015年1月至2019年5月间全国范围内一项大型前瞻性队列中的7,662名ACS患者。SHR通过以下公式计算得出:SHR = 入院血糖(mmol/L)/ [1.59×糖化血红蛋白(%)‐2.59]。主要终点是随访期间发生的主要不良心血管事件(MACE), 包括死亡, 心肌梗死和非计划性血管再通术这些合并症。次要终点是主要终点的各个组成部分。

**结果:**中位随访时间为2.1年, 发生了779例MACE事件。经多变量调整后, ACS患者中最高SHR三分位数显著与长期MACE风险增加相关(HR 1.53, 95%CI 1.24‐1.88), 与全因死亡风险增加相关(HR 1.80, 95%CI 1.29‐2.51), 以及与非计划性血管再通术风险增加相关(HR 1.44, 95%CI 1.09‐1.91)。尽管无论糖尿病患者还是非糖尿病患者, 最高SHR三分位数与MACE和全因死亡风险之间均存在显著相关性, 但这两个组别的风险模式是不同的。

**结论:**不论患者是否有糖尿病, 升高的SHR与长期结局的风险增加相关。这表明SHR是ACS后风险分层的潜在生物标志物。

## 1 INTRODUCTION

Stress hyperglycemia is a manifestation referring to transient hyperglycemia during severe illness and is common in patients who had acute coronary syndrome (ACS).^1^ Stress hyperglycemia is also reported as an independent risk factor for mortality and complications in ACS patients.^2^, ^3^, ^4^, ^5^ Most studies considered admission glucose level as the indicator of stress hyperglycemia. However, the association between elevated glucose level and clinical outcomes seemed to be more pronounced in patients without diabetes. In a meta‐analysis, nondiabetic patients with stress hyperglycemia had 3.9‐fold higher risk of in‐hospital death after myocardial infarction compared with nondiabetic patients with normoglycemia. In the setting of diabetes, the pooled risk of in‐hospital death in patients with hyperglycemia was only 1.7‐fold higher than normoglycemic subjects.^3^

Given different prognostic impacts of hyperglycemia in diabetic and nondiabetic patients, Roberts et al introduced a new marker combining the information of acute glucose level and background glycemic status, stress hyperglycemia ratio (SHR), to better identify critically ill patients with stress hyperglycemia.^6^ The superiority of SHR for short‐term risk prediction in ACS patients with or without diabetes has been confirmed by prior studies.^7^, ^8^, ^9^, ^10^ However, the association between SHR and long‐term outcomes was still controversial. Several studies reported that higher SHR was significantly associated with increased risk of adverse events, whereas some other studies indicated a nonlinear association between SHR and prognosis.^11^, ^12^, ^13^, ^14^ Moreover, the effect of diabetic status on the long‐term predictive value of SHR was also in debate. Therefore, we conducted this study to precisely evaluate the relationship between SHR and 2‐year outcomes in ACS patients using data from a large multicenter prospective cohort.

## 2 METHODS

### 2.1 Study design and population

**FIGURE 1:**
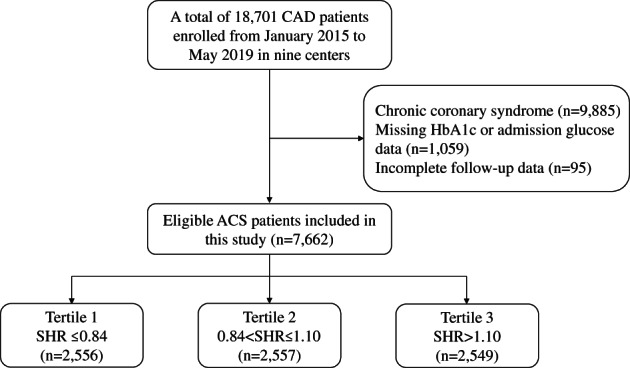
Study flow chart. ACS, acute coronary syndrome; CAD, coronary artery disease; HbA1_C_, glycosylated hemoglobin; SHR, stress hyperglycemia ratio.

Our study was a post hoc analysis of a large prospective observational nationwide cohort. From January 2015 to May 2019, a total of 18 701 adults with coronary artery disease (CAD) were recruited from Fuwai hospital (National Center for Cardiovascular Diseases) and eight other medical centers throughout China. Patients were excluded in the present analysis if (1) a diagnosis of chronic coronary syndrome was established on admission; (2) baseline data on glycosylated hemoglobin (HbA1c) or admission glucose level were missing; and (3) follow‐up data were incomplete. Finally, 7662 patients with ACS (acute myocardial infarction or unstable angina [(UA]) were included (Figure 1). These patients were further divided into three groups according to the SHR tertile: tertile 1 (SHR ≤ 0.84), tertile 2 (0.84 < SHR ≤ 1.10), and tertile 3 (SHR > 1.10).

This study complied with the Declaration of Helsinki and was approved by the institutional review committee of Fuwai Hospital. All patients signed informed consent before enrollment.

### 2.2 Data collection and definitions

Baseline data on demographic and clinical characteristics, laboratory results, and medications were collected at each clinical center by well‐trained independent clinical research coordinators. After coronary intervention was completed, details related to the procedure were recorded by two experienced interventional physicians. To assess the severity of CAD, SYNergy between percutaneous coronary intervention (PCI) with TaXus and cardiac surgery (SYNTAX) score was calculated using an online calculator (http://www.syntaxscore.com), which considered various factors related to coronary lesion data.^15^ A diagnosis of diabetes was established if diabetes history was reported in the medical records, patients had HbA1c ≥6.5% on admission or patients received antidiabetic medication currently.^16^ The average chronic glucose level (mmol/L) was estimated by HbA1c according to the formula (1.59 × HbA1c [%]−2.59). Hypertension was defined as a self‐reported hypertension history, currently receiving antihypertensive medication or consecutively measured systolic blood pressure ≥140 mmHg or/and diastolic blood pressure ≥90 mmHg for ≥3 times.^17^ Dyslipidemia was defined as fasting total cholesterol ≥5.2 mmol/L, low‐density lipoprotein cholesterol ≥3.4 mmol/L, high‐density lipoprotein cholesterol <1.0 mmol/L, triglyceride ≥1.7 mmol/L and/or receiving lipid‐lowering drugs.^18^ Medical history of prior myocardial infarction (MI), prior stroke, prior PCI, prior coronary artery bypass surgery (CABG), peripheral artery disease (PAD), chronic pulmonary disease was from self‐reported information or clinical records. Estimated glomerular filtration rate (eGFR) was calculated using the Chronic Kidney Disease Epidemiology Collaboration creatinine equation 2021.^19^ SHR was calculated according to the following equation: SHR = admission glucose (mmol/L)/(1.59 × HbA1c [%]−2.59).^6^ Blood samples from each patient were collected within 24 hours after admission and were transferred to the key laboratory of Fuwai Hospital within 3 days for testing. Admission glucose concentration was measured on a LABOSPECT 008 system (Hitachi, Tokyo, Japan). HbA1c level was measured using high‐performance liquid chromatography (Tosoh G8 HPLC Analyzer; Tosoh Bioscience, Tokyo, Japan).

### 2.3 Follow‐up and end points

Patients were followed up annually for 2 years after discharge. Data on adverse events were collected through telephone interviews, letters, or clinical visits by independent well‐trained investigators and were adjudicated centrally by two cardiologists. Primary end point was major adverse cardiovascular events (MACE), a composite of all‐cause death, MI, and unplanned revascularization. Secondary end point included individual components of the primary end point. The definition of myocardial infarction was based on the Fourth Universal Definition of Myocardial Infarction as a clinical condition characterized by signs or symptoms of newly developed myocardial ischemia with an increase in cardiac enzyme level.^20^ Unplanned revascularization included any repeat percutaneous or surgical procedures regardless of target driven by ischemic symptoms or events.

### 2.4 Statistical analysis

Continuous variables were presented as mean ± SD or median and interquartile range (if variables were skew distribution). Categorical variables were presented as frequency and percentage. one‐way analysis of variance, Kruskal‐Wallis H test, or Pearson chi‐square test were used to assess the baseline differences across SHR tertiles as appropriate. Correlations between SHR and other variables were assessed by Spearman's rank correlation coefficient. Cumulative incidences among tertiles were drawn by Kaplan–Meier curves and compared by log‐rank test. Univariable and multivariable Cox regression models were applied to identify the independent associations between SHR and MACE or all‐cause death, whereas the associations with MI and unplanned revascularization were calculated by Fine–Gray hazard models for the competing risk of death. Covariates for adjustment in multivariable model include age, sex, clinical presentation (ST‐elevation myocardial infarction [STEMI], non‐ST‐elevation myocardial infarction [NSTEMI], and UA], hypertension, diabetes, dyslipidemia, smoking history, receiving PCI, prior MI, prior stroke, prior PCI, prior CABG, PAD, chronic pulmonary disease, left ventricular ejection fraction (LVEF), body mass index, eGFR, hemoglobin, and SYNTAX score. Covariates were selected from Table 1 due to the following strategies: the variable is traditional coronary artery disease risk factors; the variable is well recognized to be associated with the risk of adverse cardiovascular events; the variable is potentially relevant to SHR status. No multicollinearity issue was found in the final model (all variance inflation factors <2.0). Restricted cubic splines analyses with 4 knots at 5th, 35th, 65th, and 95th centiles were used to present the association between continuous SHR and risk of endpoints after adjustment for covariates listed above. Receiver operating characteristic curves and Youden index were used to identify cutoff values of SHR for MACEs in patients with or without diabetes. Subgroup analyses were performed to assess the robustness of the results using interaction tests in groups stratified by age, sex, clinical presentation, primary PCI, SYNTAX score, and chronic kidney disease. For all analyses, statistical significance was defined as two‐sided *p* values <.05. Statistical analyses were performed using R version 4.2.0 (R Foundation for Statistical Computing, Vienna, Austria).

**TABLE 1:** Baseline characteristics according to SHR tertiles.

Variables	T1 (SHR ≤ 0.84) *n* = 2556	T2 (0.84 < SHR ≤ 1.10) *n* = 2557	T3 (SHR > 1.10) *n* = 2549	*p* value
SHR	0.73 ± 0.09	0.97 ± 0.08	1.37 ± 0.28	<.001
Age, years	60.7 ± 11.0	60.7 ± 11.6	61.1 ± 11.9	.332
Female	581 (22.7)	576 (22.5)	616 (24.2)	.318
BMI, kg/m^2^	25.6 ± 3.5	25.7 ± 3.4	25.7 ± 3.6	.756
SBP, mmHg	126 ± 18	128 ± 19	127 ± 18	.043
Clinical presentation, %				<.001
STEMI	833 (32.6)	1398 (54.7)	1963 (77.0)	
NSTEMI	593 (23.2)	604 (23.6)	441 (17.3)	
UA	1130 (44.2)	555 (21.7)	145 (5.7)	
Prior MI, %	418 (16.4)	367 (14.4)	337 (13.2)	.006
Prior PCI, %	474 (18.5)	476 (18.6)	427 (16.8)	.145
Prior CABG, %	43 (1.7)	42 (1.6)	37 (1.5)	.780
Prior stroke, %	360 (14.1)	340 (13.3)	381 (14.9)	.238
PAD, %	111 (4.3)	99 (3.9)	87 (3.4)	.228
COPD, %	32 (1.3)	38 (1.5)	48 (1.9)	.180
Smoking history, %	1484 (58.1)	1530 (59.8)	1548 (60.7)	.141
Diabetes, %	1009 (39.5)	989 (38.7)	1181 (46.3)	<.001
Hypertension, %	1561 (61.1)	1581 (61.8)	1572 (61.7)	.841
Dyslipidemia, %	1525 (59.7)	1547 (60.5)	1646 (64.6)	.001
Primary PCI, %	1802 (70.5)	1836 (71.8)	1965 (77.1)	<.001
SYNTAX score^a^	12 (7–20)	13 (7.5–20.5)	15 (9–22)	<.001
LVEF, %	59.8 ± 8.5	57.5 ± 8.7	55.0 ± 8.6	<.001
eGFR, ml/min/1.73m^2^	88.9 ± 17.9	90.1 ± 19.0	87.9 ± 20.4	<.001
Laboratory data				
Admission glucose, mmol/L	5.2 (4.7–6.1)	6.6 (5.9–7.8)	9.5 (7.9–12.8)	<.001
HbA1c, %	6.1 (5.7–6.9)	5.9 (5.5–6.6)	6.0 (5.5–7.3)	<.001
Hemoglobin, g/L	141 (130–152)	144 (132–154)	145 (133–156)	<.001
White blood cell, 10^9^/L	7.79 ± 2.81	8.90 ± 2.97	10.35 ± 3.53	<.001
hsCRP, mg/L	2.15 (1.05–5.70)	3.57 (1.47–9.48)	5.62 (2.26–11.49)	<.001
cTnI, ng/ml	0.01 (0.00–0.28)	0.30 (0.02–3.12)	0.96 (0.12–5.39)	<.001
CK‐MB, IU/L	11.0 (8.0–15.0)	16.0 (10.0–43.0)	21.0 (12.0–52.8)	<.001
Medications at discharge, %				
Aspirin	2466 (97.1)	2475 (97.2)	2440 (95.8)	.006
P2Y12 inhibitor	2167 (85.3)	2271 (89.2)	2338 (91.8)	<.001
ACEI/ARB	1399 (55.1)	1502 (59.0)	1629 (63.9)	<.001
Statin	2418 (95.2)	2414 (94.8)	2378 (93.3)	.008
β‐blocker	1643 (64.7)	1802 (70.7)	1904 (74.7)	<.001
Insulin	186 (14.0)	172 (13.3)	302 (23.8)	<.001
Oral hypoglycemic drugs	146 (17.1)	109 (14.8)	115 (19.7)	.066

Abbreviations: ACEI, angiotensin‐converting enzyme inhibitor; ARB, angiotensin receptor blocker; BMI, body mass index; CABG, coronary artery bypass grafting; CK‐MB, creatine kinase‐myocardial band; COPD, chronic obstructive pulmonary disease; cTnI, cardiac troponin I; eGFR, estimated glomerular filtration rate; HbA1C, glycosylated hemoglobin; hsCRP, high‐sensitivity C‐reactive protein; LVEF, left ventricular ejection fraction; MI, myocardial infarction; NSTEMI, non‐ST‐elevation myocardial infarction; PAD, peripheral artery disease; PCI, percutaneous coronary intervention; SBP, systolic blood pressure; SHR, stress hyperglycemia ratio; STEMI, ST‐elevation myocardial infarction; UA, unstable angina.

^a^ SYNTAX score was calculated using an online calculator (http://www.syntaxscore.com) by a research group blinded to the clinical data.

## 3 RESULTS

### 3.1 Baseline characteristics

A total of 7662 ACS patients (54.7% STEMI, 21.4% NSTEMI, and 23.9% UA) who met the inclusion and exclusion criteria were included in this study. Of these patients, 1773 (23.1%) were female and 3178 (41.5%) had diabetes. The mean age and SHR levels were 61.0 ± 10.6 years and 0.91 ± 0.27, respectively. Baseline characteristics across SHR tertiles are presented in Table 1. Patients with higher SHR levels had more STEMI, diabetes, and dyslipidemia, with higher SYNTAX scores and lower LVEF. They were also more likely to undergo PCI, have higher admission glucose, hemoglobin, inflammatory biomarkers (white blood cells and high‐sensitivity C‐reactive protein), myocardial biomarkers (cardiac troponin I and creatine kinase‐myocardial band), and receive more medications at discharge. Weak correlations (0.2 < *r* < 0.4) were presented between SHR and white blood cells, high‐sensitivity C‐reactive protein, cardiac troponin I, creatine kinase‐myocardial band, and LVEF (all *p* < .001) (Table S1).

### 3.2 SHR and long‐term clinical outcomes

**FIGURE 2:**
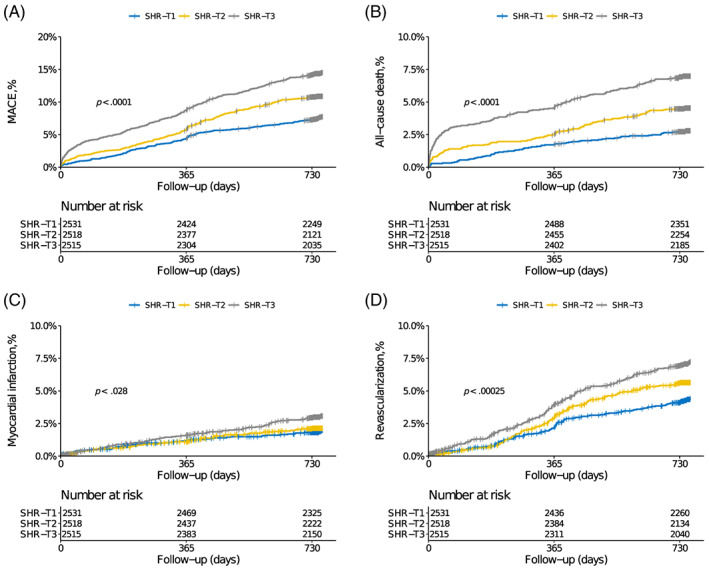
Cumulative incidences of 2‐year MACE (A), all‐cause death (B), myocardial infarction (C), and unplanned revascularization (D) according to SHR tertiles. MACE, major adverse cardiovascular events; SHR, stress hyperglycemia ratio.

During follow‐up time (median 2.1 years, interquartile range: 2.0–2.1 years), 779 MACE events (10.2%) and 349 death events (4.6%) occurred, with response rates of 98.7% at first year and 97.1% at second year. Cumulative incidences of clinical outcomes across SHR tertiles are illustrated in Figure 2. Stepwise higher rates of MACE and each component were observed in patients with higher SHR (Table 2). In the multivariable analysis, the highest tertile of SHR was significantly associated with higher risks of MACE (HR 1.53, 95% CI 1.24–1.88), all‐cause death (HR 1.80, 95% CI 1.29–2.51), and unplanned revascularization (HR 1.44, 95% CI 1.09–1.91). Results were similar when SHR was calculated on a continuous scale (Table 3 and Figure S1).

**TABLE 2:** Incidence of clinical outcomes across SHR tertile groups at 2 years.

Variables	T1 (SHR ≤ 0.84) *n* = 2556	T2 (0.84 < SHR ≤ 1.10) *n* = 2557	T3 (SHR > 1.10) *n* = 2549	*p* value
MACE, %	184 (7.2)	258 (10.1)	337 (13.2)	<.001
All‐cause death, %	69 (2.7)	111 (4.3)	169 (6.6)	<.001
Myocardial infarction, %	47 (1.8)	52 (2.0)	72 (2.8)	.04
Revascularization, %	105 (4.1)	134 (5.2)	165 (6.5)	<.001
Follow‐up (years)	2.01 ± 0.23	1.99 ± 0.30	1.95 ± 0.40	<.001

Abbreviations: MACE, major adverse cardiovascular events; SHR: stress hyperglycemia ratio.

**TABLE 3:** Stress hyperglycemia ratio and long‐term adverse outcomes.

Variables	Crude HR	*p* value	Adjusted HR	*p* value
MACE				
T1	Reference		Reference	
T2	1.43 (1.18–1.73)	<.001	1.32 (1.07–1.62)	.008
T3	1.83 (1.54–2.17)	<.001	1.53 (1.24–1.88)	<.001
Per 1 unit increase	2.09 (1.77–2.47)	<.001	1.57 (1.29–1.92)	<.001
All‐cause death				
T1	Reference		Reference	
T2	1.63 (1.21–2.21)	<.001	1.44 (1.02–2.03)	.037
T3	2.53 (1.91–3.35)	<.001	1.80 (1.29–2.51)	<.001
Per 1 unit increase	2.74 (2.22–3.38)	<.001	1.72 (1.32–2.25)	<.001
Myocardial infarction				
T1	Reference		Reference	
T2	1.28 (0.84–1.96)	.250	1.18 (0.74–1.87)	.490
T3	1.78 (1.20–2.64)	.004	1.48 (0.93–2.36)	.100
Per 1 unit increase	1.59 (1.11–2.27)	.011	1.18 (0.77–1.82)	.440
Revascularization				
T1	Reference		Reference	
T2	1.38 (1.06–1.79)	.017	1.24 (0.95–1.63)	.120
T3	1.69 (1.31–2.17)	<.001	1.44 (1.09–1.91)	.011
Per 1 unit increase	1.65 (1.32–2.06)	<.001	1.44 (1.10–1.89)	.008

*Note*: Adjusted for age, sex, clinical presentation, prior percutaneous coronary intervention, prior coronary artery bypass grafting, prior myocardial infarction, prior stroke, peripheral artery disease, chronic pulmonary disease, smoking history, hypertension, diabetes, dyslipidemia, body mass index, estimated glomerular filtration rate, hemoglobin, percutaneous coronary intervention, SYNTAX score, left ventricular ejection fraction.

Abbreviations: HR, hazard ratio; MACE, major adverse cardiovascular events.

### 3.3 Clinical outcomes stratified by diabetes status

**FIGURE 3:**
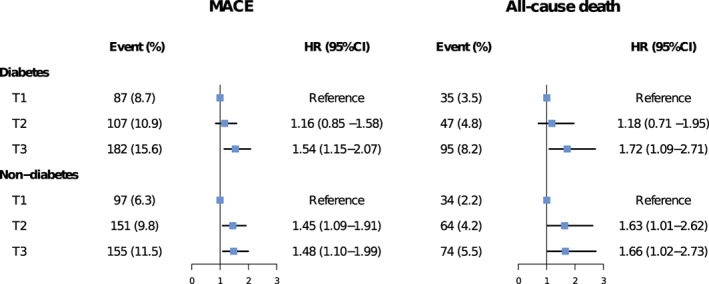
Adjusted risk of MACE and all‐cause death across SHR tertiles in patients with diabetes and without diabetes. CI, confidence interval; HR, hazard ratio; MACE, major adverse cardiovascular events; SHR, stress hyperglycemia ratio.

**FIGURE 4:**
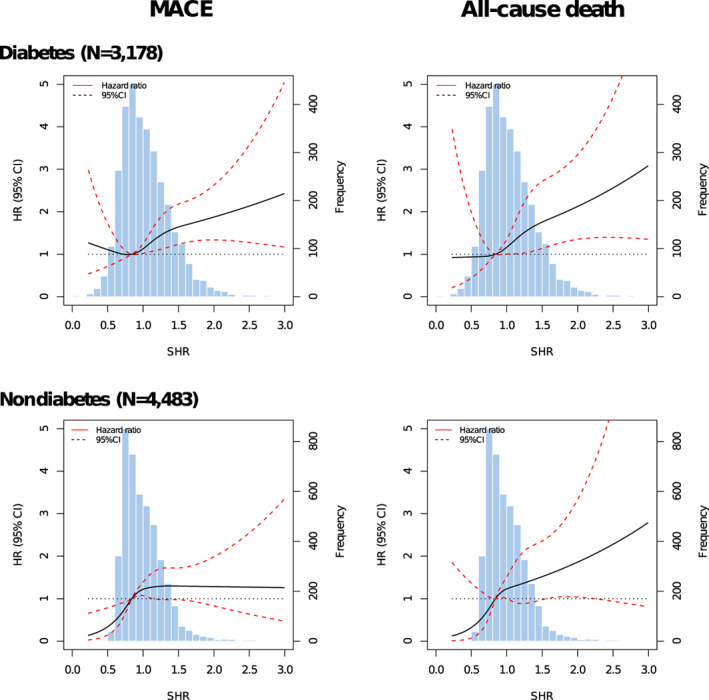
SHR on a continuous scale and adjusted risk of MACE and all‐cause death in patients with and without diabetes. Hazard ratio is depicted by black solid line and 95% confidence intervals are depicted by red dotted lines. Distribution of SHR (blue blocks) is presented as histogram. Reference value was defined as 0.84 (the first tertile). Multivariable adjustment was for age, sex, clinical presentation, hypertension, dyslipidemia, smoking history, receiving PCI, prior myocardial infarction, prior stroke, prior PCI, prior coronary artery bypass surgery, peripheral artery disease, chronic pulmonary disease, left ventricular ejection fraction, body mass index, estimated glomerular filtration rate, hemoglobin, and SYNTAX score. CI, confidence interval; HR, hazard ratio; MACE, major adverse cardiovascular events; PCI, percutaneous coronary intervention; SHR, stress hyperglycemia ratio.

Figure 3 presents the associations between SHR tertiles and adjusted risks of MACE and all‐cause death in patients with or without diabetes. In patients with diabetes, only the third SHR tertile (SHR >1.10) significantly predicted the risks of MACE (HR 1.54, 95% CI 1.15–2.07) and all‐cause death (HR 1.72, 95% CI 1.09–2.71), whereas both the second (0.84 < SHR ≤ 1.10) and third tertiles (SHR > 1.10) were significantly associated with risks of MACE (T2 vs. T1: HR 1.45, 95% CI 1.09–1.91; T3 vs. T1: HR 1.48, 95% CI 1.10–1.99) and all‐cause death (T2 vs. T1: HR 1.63, 95% CI 1.01–2.62; T3 vs. T1: HR 1.66, 95% CI 1.02–2.73) in subjects without diabetes. No significant interaction effect between SHR and diabetes status was observed for the risk of adverse outcomes (MACE: *p* for interaction = .396; all‐cause death: *p* for interaction = .499). The third SHR tertile was not significantly associated with the risk of MI and unplanned revascularization in patients with or without diabetes (Figure S2). Patterns illustrating associations between SHR on continuous scales and end points using restricted cubic splines are presented in Figure 4. Association between SHR and MACE appeared to be the J‐shaped curve in diabetic patients. However, the pattern was different in the nondiabetes group. Higher SHR was significantly associated with increased MACE risk, and then the association plateaued above the SHR threshold of around 1.00. In terms of all‐cause death, patterns of diabetes and nondiabetes groups were similar but blunter.

### 3.4 SHR cutoff values and subgroup analysis

**FIGURE 5:**
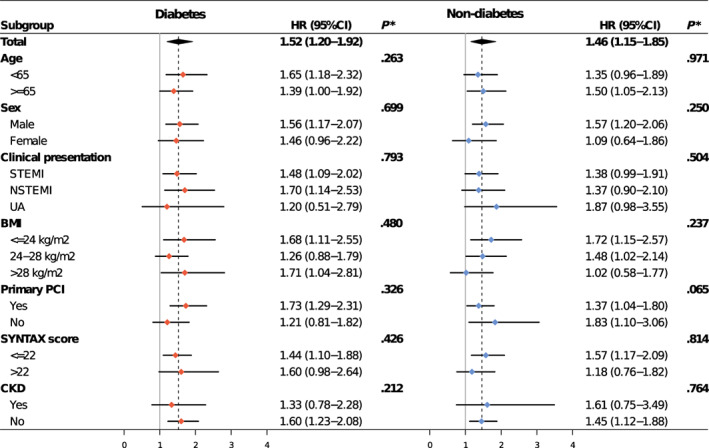
Subgroup analysis of primary endpoint according to the cutoff SHR values in patients with diabetes and without diabetes. BMI, body mass index; CI, confidence interval; CKD, chronic kidney disease; HR, hazard ratio; NSTEMI, non‐ST‐elevation myocardial infarction; PCI, percutaneous coronary intervention; STEMI, ST‐elevation myocardial infarction; UA, unstable angina. *p indicated *p* value for interaction.

The optimal cutoff values of SHR for MACE prediction based on Youden index were 1.02 for patients with diabetes and 0.92 for patients without diabetes (Figure S3). Compared with SHR below cutoff values, adjusted MACE risk associated with SHR above cutoff values was 1.52 (95% CI 1.20–1.92) in the diabetes group and 1.46 (95% CI 1.15–1.85) in the nondiabetes group (Figure 5). The results remained consistent across subgroups (All *p* for interaction >.05) (Figure 5).

## 4 DISCUSSION

In this study of 7662 ACS patients from a prospective multicenter cohort, we found that subjects with high SHR exhibited a lower LVEF, and elevated levels of inflammatory and myocardial biomarkers. high SHR levels were independently associated with increased risk of MACE, all‐cause death, and unplanned revascularization. Associations between high SHR levels and risk of MACE and death remained positive but with different risk patterns in patients with or without diabetes. The prognostic value of SHR were consistent across subgroups.

Stress hyperglycemia, secondary to neuroendocrine derangement affected by severe illness, was found to be associated with poor prognosis in patients with ACS.^1^ Admission glucose level was the most common metric of stress hyperglycemia. However, its prognostic effect was questioned in those with established diabetes. An early meta‐analysis reported that acute MI (AMI) patients without diabetes had a greater hyperglycemia‐associated risk of in‐hospital death with lower glucose thresholds compared with those with diabetes.^3^ Similarly, according to a national analysis of 141 680 elderly AMI patients, higher glucose levels were significantly associated with both short‐term and long‐term risk of mortality in patients without known diabetes, whereas mortality risk started to rise when glucose was higher than 240 mg/dL in those with established diabetes.^2^ This result was confirmed by other studies.^8^, ^21^ One possible explanation is that the high admission glucose level was derived from both stress and background glycemic status in diabetic patients, thus admission glucose might exaggerate the degree of stress in this population.

To eliminate the influence of chronic glycemic level, SHR, calculated by admission glucose and estimated chronic glucose, was introduced as a new metric of stress‐induced hyperglycemia. In a study of 1553 AMI patients, Marenzi et al reported that SHR was independently associated with in‐hospital adverse outcomes and was a better prognostic predictor compared with admission glucose, especially in patients with diabetes. Another study of 474 diabetic patients after AMI indicated that SHR showed a better prognostic value in acute kidney injury than admission glucose alone.^9^ Similar results were also found in the study with a larger sample size or posthoc analysis of a randomized clinical trial.^7^, ^22^ However, it is questioned whether SHR was a reliable predictor of long‐term outcomes. A large‐scale study conducted by Sia et al showed the independent association between SHR and 1‐year mortality in STEMI patients regardless of diabetic status and the superiority of SHR over admission glucose in 1‐year mortality prediction. In contrast, according to a study of 6287 STEMI patients with 5‐year follow‐up, the association between the highest quartiles and risk of long‐term worse prognosis became significant only in nondiabetic patients but not in diabetic patients.^13^ As SHR was categorized into only two groups based on quartiles or cutoff points in these studies, the potential nonlinear relationship between SHR and long‐term outcomes might be neglected. Two recent studies conducted in ACS patients both indicated that the association between SHR and long‐term adverse outcomes was nonlinear, presenting as U‐shaped or J‐shaped curves, but interestingly, was more pronounced in diabetic patients.^12^, ^14^ However, a largely linear association between SHR and long‐term survival was presented in a recent study of 2089 AMI patients conducted by Luo et al.^23^ Compared with prior studies, we performed a comprehensive analysis on the long‐term prognostic impact of SHR. Although the risk patterns of SHR were influenced by diabetic status, high SHR (defined as the highest SHR tertile or above the cutoff values) was significantly associated with higher rates of adverse events in patients with and without diabetes even after multivariable adjustment. Our study indicated that SHR was a reliable marker in long‐term risk stratification among ACS patients and might help identify high‐risk patients who could benefit from glucose‐lowing therapy.

It is still uncertain whether stress‐induced hyperglycemia has direct effects on adverse outcomes in ACS patients, as there is little evidence from clinical trials supporting the survival benefits associated with intensive insulin treatment in patients after ACS. Although DIGAMI (Diabetes Mellitus Insulin Glucose Infusion in AMI) trials reported that patients receiving insulin infusion had significantly lower rates of death compared with control groups, other studies including DIGAMI 2, HT‐5 (Hyperglycemia: Intensive Insulin Infusion in Infarction), and BIOMArCS‐2 (Randomized Trial to Evaluate the Clinical Value of Intensive Glucose Monitoring and Regulation in Myocardial Infarction) trials failed to find a persistent beneficial effect of intensive glucose‐lowing therapy.^24^, ^25^, ^26^, ^27^, ^28^ However, multiple experimental and clinical studies demonstrated that acute hyperglycemia could exert a direct contribution to acute or chronic implications through various mechanisms. Acute glucose fluctuation induces exacerbated oxidative stress, which plays a central role in tissue damage and chronic complication.^1^, ^29^, ^30^ Moreover, acute hyperglycemia triggered endothelial dysfunction, vascular inflammation, and coagulation activation, leading to the deterioration of myocardial injury, which is concordant with our findings about the correlations between inflammatory and myocardial injury markers and SHR.^31^, ^32^, ^33^, ^34^ High heterogeneity across clinical trials might be a plausible explanation for the conflicting clinical results, as apparent differences in patient populations and glucose targets exist in these trials. Additionally, some evidence demonstrates that chronic hyperglycemia could alleviate the damage caused by acute hyperglycemia, hence the effect of chronic glucose level should be taken into consideration when evaluating the efficacy of intensive glucose‐lowing treatment.^35^, ^36^ In a posthoc analysis of HT‐5 data, SHR seemed to be a better prognostic indicator during glucose‐lowing therapy compared with conventional mean glucose level.^7^ Therefore, it is suggested that a new glucose target derived from SHR be applied in further glucose‐lowing clinical trials.

Our study also had several limitations. First, due to its observational nature, although numerous covariates were adjusted in the model, potential unmeasured confounders could still influence the results. Second, as we included only patients from China, the generalizability of our findings was limited to other ethnic populations. Third, data on glucose‐lowing therapy at discharge were incomplete in our study. Furthermore, glucose fluctuation and management of hyperglycemia during hospital stay and follow‐up were unavailable, which may be the source of bias. Finally, the definition of diabetes was not precise, for we did not distinguish type 1 and type 2 diabetes, and also did not include new‐onset diabetes due to a lack of data on oral glucose tolerance tests.

## 5 CONCLUSION

In this large nationwide cohort, our results suggest that high SHR was independently associated with long‐term poor prognosis irrespective of diabetic status, indicating that SHR is a reliable biomarker for risk stratification. Further study are warranted to confirm the results.

## AUTHOR CONTRIBUTIONS

Jinqing Yuan, Yaling Han, and Guyu Zeng contributed to the conception and design of the work. Ying Song, Zheng Zhang, Jingjing Xu, Zhenyu Liu, Xiaofang Tang, XiaoZeng Wang, Yan Chen, Yongzhen Zhang, Pei Zhu, Xiaogang Guo, Lin Jiang, Zhifang Wang, Ru Liu, Qingsheng Wang, Yi Yao, and Yingqing Feng contributed to data collection and analysis. Guyu Zeng drafted the manuscript. Jinqing Yuan and Yaling Han critically revised the manuscript. All authors read and approved the final manuscript.

## FUNDING INFORMATION

This study was supported by The National Key Research and Development Program of China (grand No. 2016YFC1301300 and 2016YFC1301301), the National Clinical Research Center for Cardiovascular Disease, Fuwai Hospital, Chinese Academy of Medical Sciences (grand No. NCRC2020013) and Chinese Academy of Medical Sciences Innovation Fund for Medical Sciences (grand No. 2020‐I2M‐C&T‐B‐049).

## CONFLICT OF INTEREST STATEMENT

The authors declare no conflict of interest.

## Supporting information


**Table S1.** Collinearity analysis of covariates.
**Table S2.** Spearman correlation analysis between stress hyperglycemia ratio and other variables.
**Table S3.** The association between admission glucose, HbA1c, and long‐term major adverse cardiovascular events (MACE).
**Table S4.** C‐statistic of stress hyperglycemia ratio.
**Figure S1.** SHR on a continuous scale and adjusted risk of major adverse cardiovascular events and all‐cause death in the entire population.
**Figure S2.** Adjusted risk of myocardial infarction and unplanned revascularization across SHR tertiles in patients with diabetes and without diabetes. CI, confidence interval; HR, hazard ratio; SHR, stress hyperglycemia ratio.
**Figure S3.** Receiver operating characteristic curves of stress hyperglycemia ratio for prediction of major adverse cardiovascular events in patients with diabetes and without diabetes.
