# Supplementary material for: Stress hyperglycemia ratio and long‐term prognosis in patients with acute coronary syndrome: A multicenter, nationwide study
Source: J Diabetes. 2023 May 3;15(7):557–68. doi: 10.1111/1753-0407.13400 (PMC10345977; doi:10.1111/1753-0407.13400)
Supplement: Supplementary file 1 — Table S1. Collinearity analysis of covariates. Table S2. Spearman correlation analysis between stress hyperglycemia ratio and other variables. Table S3. The association between admission glucose, HbA1c, and long‐term major adverse cardiovascular events (MACE). Table S4. C‐statistic of stress hyperglycemia ratio. Figure S1. SHR on a continuous scale and adjusted risk of major adverse cardiovascular events and all‐cause death in the entire population. Figure S2. Adjusted risk of myocardial infarction and unplanned revascularization across SHR tertiles in patients with diabetes and without diabetes. CI, confidence interval; HR, hazard ratio; SHR, stress hyperglycemia ratio. Figure S3. Receiver operating characteristic curves of stress hyperglycemia ratio for prediction of major adverse cardiovascular events in patients with diabetes and without diabetes. [file JDB-15-557-s001.docx]

Supplemental Materials

Table S1. Collinearity analysis of covariates.

|  | Collinearity statistics |  |
| --- | --- | --- |
| Covariates | Tolerance | Variance inflation factors |
| Sex | 0.69 | 1.45 |
| Age | 0.58 | 1.71 |
| Clinical presentation | 0.70 | 1.43 |
| Hypertension | 0.91 | 1.10 |
| Diabetes | 0.94 | 1.06 |
| Dyslipidemia | 0.90 | 1.11 |
| Smoking history | 0.78 | 1.29 |
| Receiving PCI | 0.92 | 1.09 |
| Prior MI | 0.79 | 1.26 |
| Prior stroke | 0.95 | 1.05 |
| Prior PCI | 0.82 | 1.22 |
| Prior CABG | 0.92 | 1.08 |
| PAD | 0.97 | 1.03 |
| COPD | 0.99 | 1.01 |
| LVEF | 0.78 | 1.29 |
| BMI | 0.87 | 1.15 |
| eGFR | 0.66 | 1.51 |
| Hemoglobin | 0.78 | 1.29 |
| SYNTAX score | 0.88 | 1.19 |
| SHR | 0.84 | 1.19 |

Abbreviations: BMI, body mass index; CABG, coronary artery bypass grafting; COPD, chronic obstructive pulmonary disease; eGFR, estimated glomerular filtration rate; HbA1C, glycosylated hemoglobin; LVEF, left ventricular ejection fraction; PAD, peripheral artery disease; MI, myocardial infarction; PCI, percutaneous coronary intervention; SHR, stress hyperglycemia ratio.

Table S2. Spearman correlation analysis between SHR and other variables.

|  | Correlation coefficients |  |
| --- | --- | --- |
|  | Rho | P value |
| eGFR, ml/min/1.73m2 | 0.070 | <0.001 |
| Age, y | -0.022 | 0.004 |
| BMI, kg/m2 | 0.009 | 0.219 |
| SBP, mmHg | -0.023 | 0.007 |
| White blood cells, 10^9^/L | 0.266 | <0.001 |
| hsCRP, mg/L | 0.237 | <0.001 |
| cTnI, ng/ml | 0.387 | <0.001 |
| CK-MB, IU/L | 0.302 | <0.001 |
| LVEF,% | -0.218 | <0.001 |
| SYNTAX score | 0.088 | <0.001 |
| Hemoglobin, g/L | 0.082 | <0.001 |

Table S3. The association between admission glucose, HbA1c and long-term major adverse cardiovascular events (MACE).

|  | Crude HR (95%CI) | P value | Adjusted HR (95%CI) | P value |
| --- | --- | --- | --- | --- |
| Admission glucose (mmol/L) |  |  |  |  |
| T1 (≤5.94) | Reference |  | Reference |  |
| T2 (5.94-8.03) | 1.46 (1.20-1.78) | **<0.001** | 1.26 (1.02-1.57) | **0.035** |
| T3 (>8.03) | 2.28 (1.90-2.73) | **<0.001** | 1.78 (1.41-2.23) | **<0.001** |
| Per 1 unit increase | 1.09 (1.07-1.10) | **<0.001** | 1.06 (1.04-1.08) | **<0.001** |
| HbA1c, % |  |  |  |  |
| T1 (≤5.7) | Reference |  | Reference |  |
| T2 (5.7-6.5) | 1.14 (0.95-1.36) | 0.165 | 0.93 (0.77-1.13) | 0.486 |
| T3 (>6.5) | 1.52 (1.28-1.80) | **<0.001** | 1.29 (0.95-1.76) | 0.105 |
| Per 1 unit increase | 1.06 (1.04-1.08) | **<0.001** | 1.03 (1.00-1.07) | **0.033** |

Adjusted for age, sex, clinical presentation, prior percutaneous coronary intervention, prior coronary artery bypass grafting, prior myocardial infarction, prior stroke, peripheral artery disease, chronic pulmonary disease, smoking history, hypertension, diabetes, dyslipidemia, body mass index, estimated glomerular filtration rate, hemoglobin, percutaneous coronary intervention, SYNTAX score, left ventricular ejection fraction.

HR, hazard ratio.

Table S4. C-statistic of stress hyperglycemia ratio

|  | C-statistics (95%CI) | ΔC-statistics (95%CI) | P value |
| --- | --- | --- | --- |
| Original model | 0.686 (0.665, 0.708) | -0.006 (-0.010, -0.002) | 0.007 |
| Original model +SHR | 0.692 (0.671, 0.713) | Reference |  |
| Original model +HbA1c | 0.689 (0.668, 0.710) | -0.003 (-0.008, 0.002) | 0.180 |
| Original model +admission glucose | 0.696 (0.676, 0.717) | 0.004 (-0.001,0.009) | 0.079 |

Original model included age, sex, clinical presentation, prior percutaneous coronary intervention, prior coronary artery bypass grafting, prior myocardial infarction, prior stroke, peripheral artery disease, chronic pulmonary disease, smoking history, hypertension, diabetes, dyslipidemia, body mass index, estimated glomerular filtration rate, hemoglobin, percutaneous coronary intervention, SYNTAX score, left ventricular ejection fraction.

CI, confidence interval; SHR, stress hyperglycemia ratio.


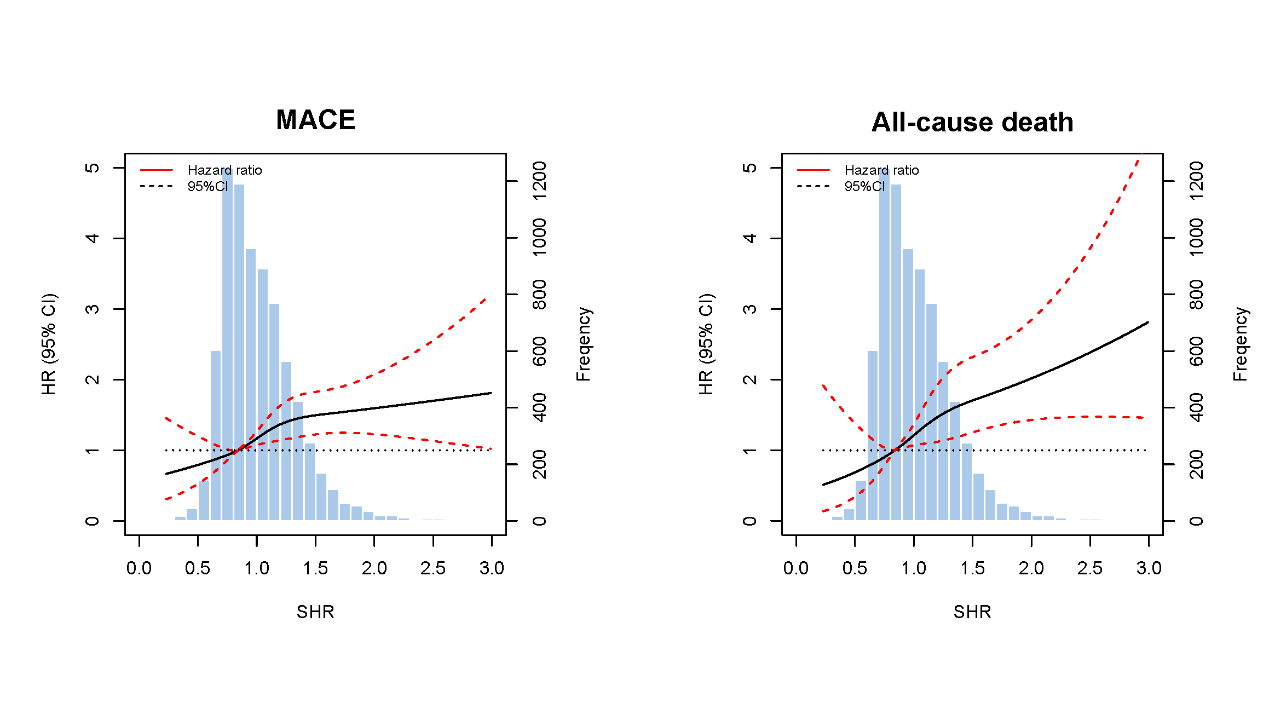


Figure S1. SHR on a continuous scale and adjusted risk of MACE and all-cause death in the entire population.

Hazar ratio is depicted by black solid line and 95% confidence intervals are depicted by red dotted lines. Distribution of SHR (blue blocks) is presented as histogram. Reference value was defined as 0.84 (the first tertile). Multivariable adjustment was for age, sex, clinical presentation, hypertension, dyslipidemia, smoking history, receiving PCI, prior MI, prior stroke, prior PCI, prior coronary artery bypass surgery, peripheral artery disease, chronic pulmonary disease, left ventricular ejection fraction, body mass index, estimated glomerular filtration rate, hemoglobin and SYNTAX score. CI, confidence interval; HR, hazard ratio; MACE, major adverse cardiovascular events; SHR stress hyperglycemia ratio.


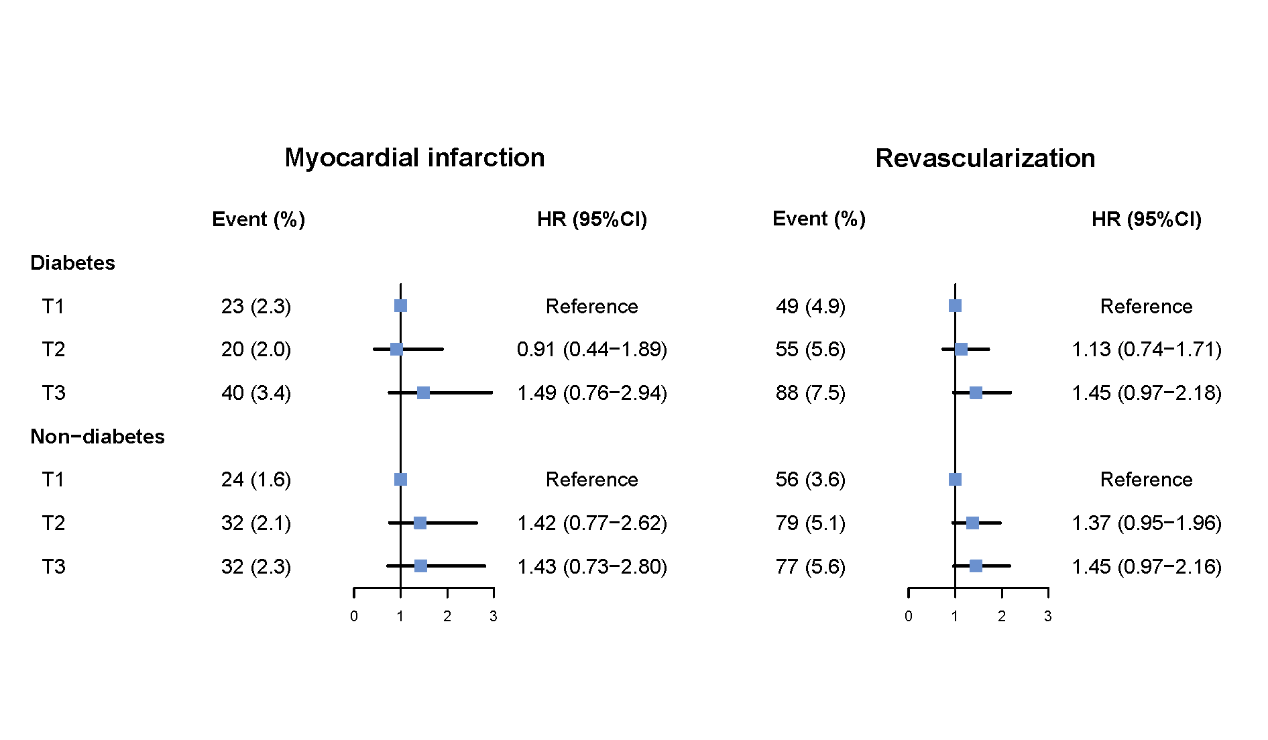


Figure S2. Adjusted risk of myocardial infarction and unplanned revascularization across SHR tertiles in patients with diabetes and without diabetes. CI, confidence interval; HR, hazard ratio; SHR stress hyperglycemia ratio.


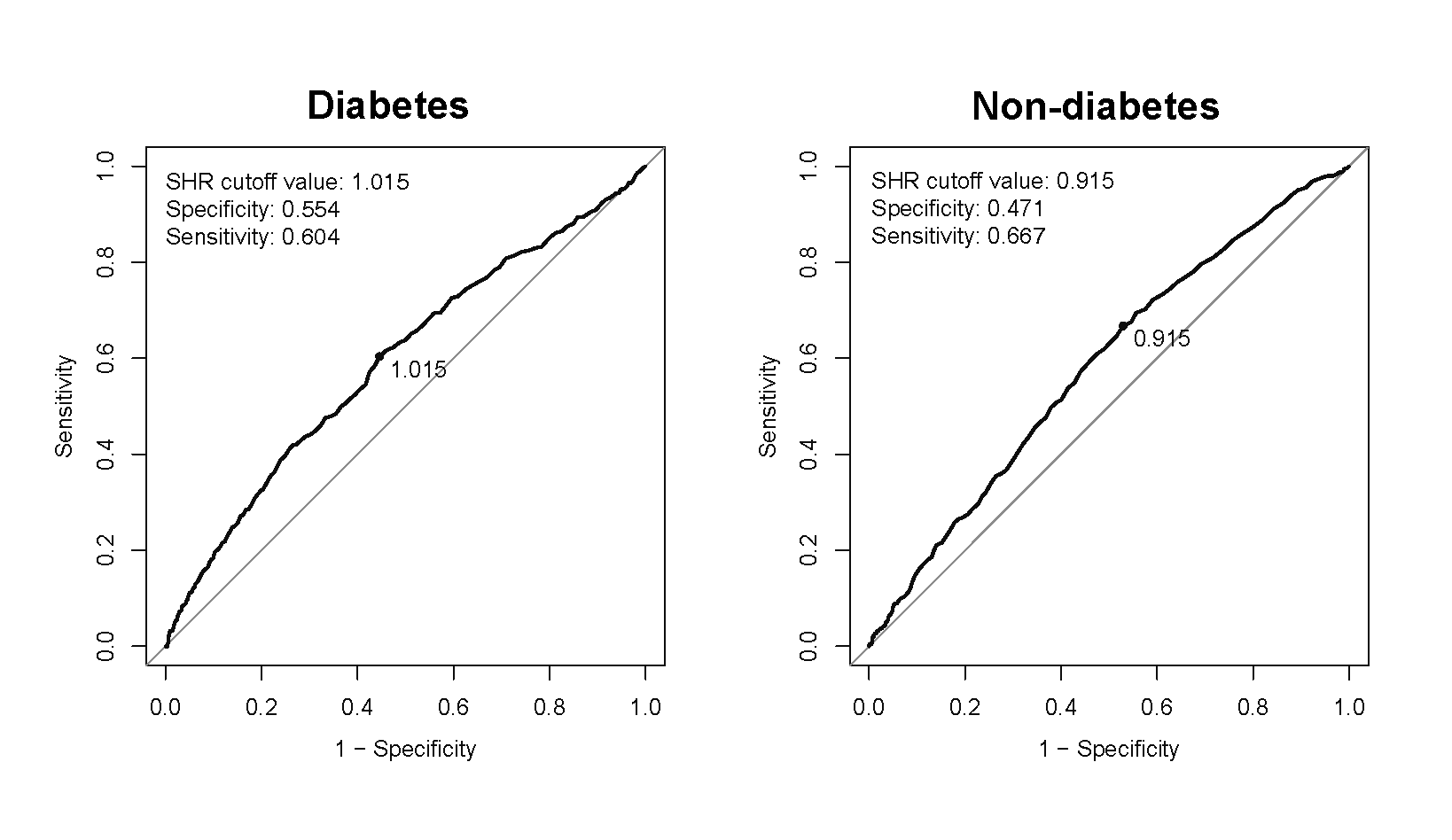
 Figure S3. Receiver operating characteristic curves of SHR for prediction of major adverse cardiovascular events in patients with diabetes and without diabetes.
